# The Role of Crowding Forces in Juxtaposing β-Globin Gene Domain Remote Regulatory Elements in Mouse Erythroid Cells

**DOI:** 10.1371/journal.pone.0139855

**Published:** 2015-10-05

**Authors:** Arkadiy K. Golov, Alexey A. Gavrilov, Sergey V. Razin

**Affiliations:** 1 Institute of Gene Biology of the Russian Academy of Sciences, Vavilov Street 34/5, 119334, Moscow, Russia; 2 Molecular Biology Department, Biological Faculty, Lomonosov Moscow State University, 119992, Moscow, Russia; Laval University Cancer Research Centre, CANADA

## Abstract

The extremely high concentration of macromolecules in a eukaryotic cell nucleus indicates that the nucleoplasm is a crowded macromolecular solution in which large objects tend to gather together due to crowding forces. It has been shown experimentally that crowding forces support the integrity of various nuclear compartments. However, little is known about their role in control of chromatin dynamics in vivo. Here, we experimentally addressed the possible role of crowding forces in spatial organization of the eukaryotic genome. Using the mouse β-globin domain as a model, we demonstrated that spatial juxtaposition of the remote regulatory elements of this domain in globin-expressing cells may be lost and restored by manipulation of the level of macromolecular crowding. In addition to proving the role of crowding forces in shaping interphase chromatin, our results suggest that the folding of the chromatin fiber is a major determinant in juxtaposing remote genomic elements.

## Introduction

It has become increasingly evident that an apparent order in the eukaryotic cell nucleus originates out of disorder because of the self-assembly of functional nuclear compartments, which can be initiated by various “seeding events” including realization of functional processes [1–6]. The integrity of these compartments (such as nucleoli, Cajal bodies, and PML bodies) is supported by weak specific interactions and non-specific entropic forces (in particular, by “depletion attraction forces” [4, 7, 8]) that originate in crowded macromolecular solutions [9–12]. Under conditions of macromolecular crowding, large objects, including various macromolecular complexes, tend to gather together due to the bombardments by smaller molecules that are in a constant state of Brownian motion. In a mixture of different-sized particles, aggregation of large particles would result in an overlap of excluded volumes surrounding these particles and thus in an increase of the accessible space for smaller particles ensuring a gain in entropy [4, 7, 9, 11, 13, 14]. It has been experimentally shown that entropic forces play an important role in maintaining the integrity of nucleoli and certain nuclear bodies [15]. More precisely, it has been shown that under hypotonic conditions, expansion of the nucleus correlates with disappearance of nucleoli, Cajal bodies and PML bodies. However, these compartments were reestablished when an inert crowding agent (polyethylene glycol) was added to the suspension of the nuclei in a hypotonic solution.

Recent evidence suggests that the spatial organization of the genome reflects its activity and is tightly connected with the functional compartmentalization of the cell nucleus [16–20]. Using the chromosome conformation capture (3C) experimental procedure, de Laat and collaborators have demonstrated that in mouse erythroid cells, the promoters of β-globin genes are positioned in proximity to the locus control region (LCR) in an expression-dependent fashion [21]. It has been proposed that the promoters of transcribed globin genes and remote regulatory elements are assembled in a common activating complex, which is termed the active chromatin hub (ACH) [22, 23]. Other observations suggest that enhancers and target promoters are juxtaposed in a restricted nuclear compartment where they retain a certain degree of mobility [24, 25]. Whatever the particular mode of juxtaposing enhancers and promoters, it is clear that chromatin is organized in various loops within a complex 3D network [16, 23, 26, 27]. The ends of chromatin loops are likely held together by protein-protein interactions (such as dimerization/oligomerization of proteins bound to enhancers and promoters). However, these interactions are relatively weak, and the stability of the complexes may depend on the contribution of depletion attraction forces [4]. Here, we checked if the depletion attraction forces contribute to the proximity retention of the remote genomic elements constituting the mouse β-globin domain ACH. Using cultured mouse erythroid cells as a model, we demonstrated that a decrease in the level of macromolecular crowding results in a significant loss of interaction between the components of β-globin domain ACH, which is indicated by a decrease in the level of the 3C signal. With the increase in the level of macromolecular crowding, the juxtaposition of the ACH components was reestablished.

## Results and Discussion

**Fig 1:**
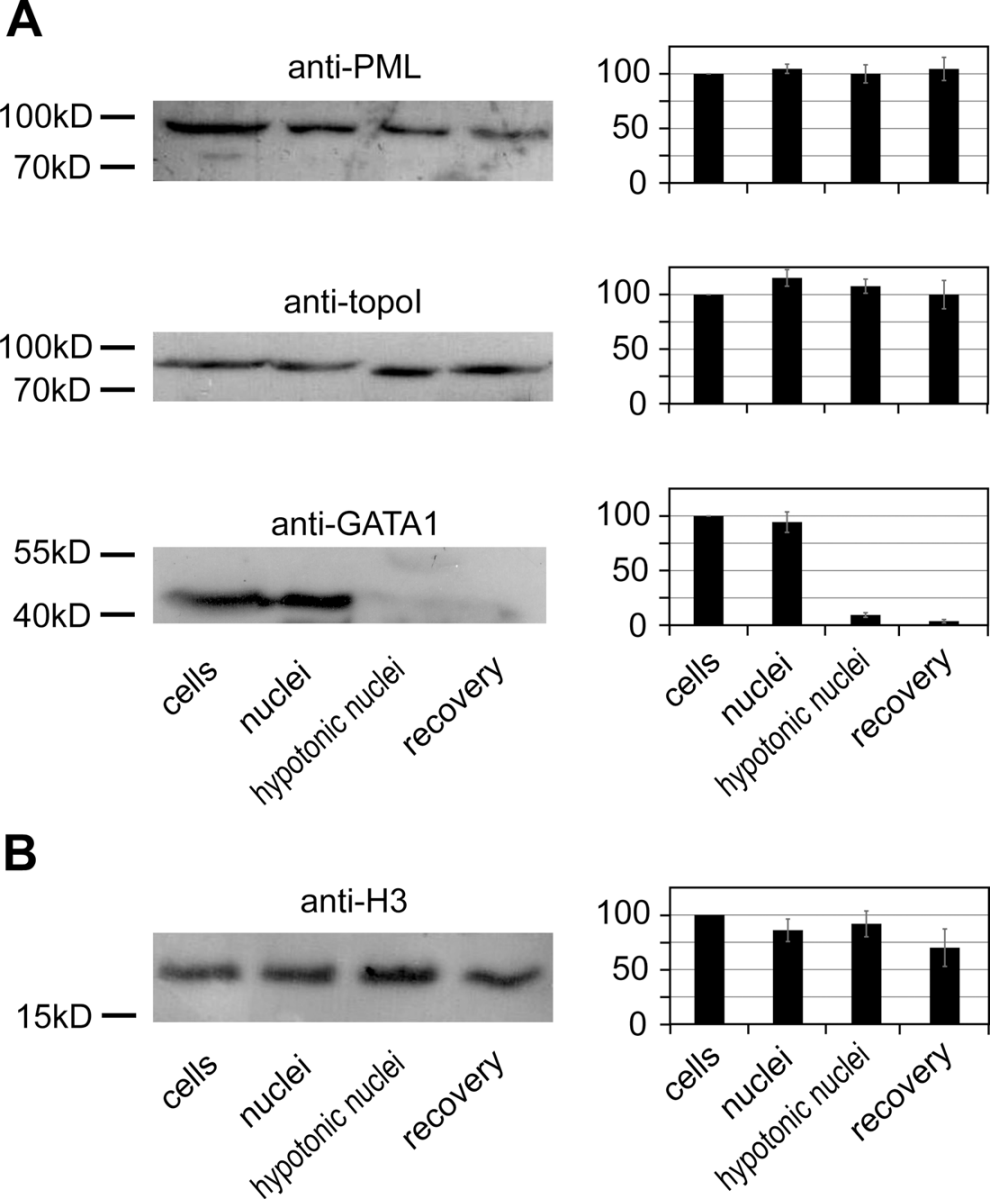
Retention of PML, DNA topoisomerase I, GATA–1, and histone H3 in nuclei after cell lysis and subsequent treatments (Western blot analysis). Representative images of Western blots are shown at the left part of the Figure, and the results of the analysis at the right part of the Figure. The positions of size markers are indicated to the left of the blots. The intensities of bands were determined using ImageJ software. In experiments shown in (A) the determined intensities of each band were normalized to the intensity of the histone H3 band in the same sample (section B). Diagrams show the average results of three independent experiments. The amount of protein in non-lysed cells was considered as 100%. The error bars represent SEM.

In all of the experiments, we used induced (expressing globin genes) cultured mouse erythroid cells (murine erythroleukemia cells, MEL). This model was chosen because we aimed to study the configuration of the β-globin ACH. The original protocol for the isolation of nuclei in isotonic buffer and subsequent expansion in hypotonic buffer [15] was slightly modified to the work with MEL cells (see Methods section for details). We first checked if we could reproduce with our experimental model the reversible disassembly of nuclear compartments as a result of a decrease in the level of macromolecular crowding. With this aim, we used the experimental strategy described by R. Hancock [15]. MEL cells were gently lysed by digitonin in an isotonic solution. Next, the nuclei were expanded in a hypotonic buffer and then returned to the initial size by the addition of an inert crowding agent (PEG 8 kDa). It has previously been reported that, after cell lysis with digitonin, the nuclear envelope retains intranuclear proteins and the integrity of nuclear compartments is not compromised [15, 28]. Small molecules, including some proteins, can pass though nuclear pores and thus will be partially or even fully lost from isolated nuclei in the absence of cytoplasm. However, the average molecular weight of diffusely localized proteins present in nucleoplasm is 68 kDa [29], and proteins of this size cannot freely pass through nuclear pores [15] and thus are retained in nuclei. To check if this is indeed the case, we determined what portion of GATA–1 (43 kDa), DNA topoisomerase I (91 kDa) and PML (98 kDa) remained in the nuclei after cell lysis and subsequent treatments (see below). With this aim equal aliquots were taken after each treatment, nuclei were precipitated and, after protein separation by SDS-PAGE and Western blotting, the above-mentioned proteins were immuno-stained. Histone H3 was used as a loading control to make a correction for the loss of material in the course of experimental manipulations. This protein is tightly bound to DNA and for this reason can hardly be solubilized in the course of cell lysis and hypotonic treatment of isolated nuclei. The results presented in Fig 1 demonstrated that ~90% of GATA–1 was retained in the nuclei after cell lysis by digitonin. The retention of DNA topoisomerase I and PML was almost complete. It is also of note that the cell nucleus is crowded with macromolecules of various nature including DNA (in the form of chromatin) and RNA which is complexed with different proteins. Both chromatin and RNP particles are retained in isolated nuclei. Thus, there are good reasons to believe that the level of macromolecular crowding in nuclei isolated using digitonin treatment under isotonic conditions closely matches that in nuclei within cells as long as the membrane is not damaged and the nuclear volume remains unchanged. The subsequent expansion of nuclei in hypotonic buffer would result in redistribution of macromolecules in a larger volume, with a consequent decrease of the level of macromolecular crowding [15]. A loss of some relatively small nuclear proteins in the course of hypotonic treatment (as has been observed for GATA–1; Fig 1) also contributes to the decrease of the level of macromolecular crowding in hypotonic nuclei.

**Fig 2:**
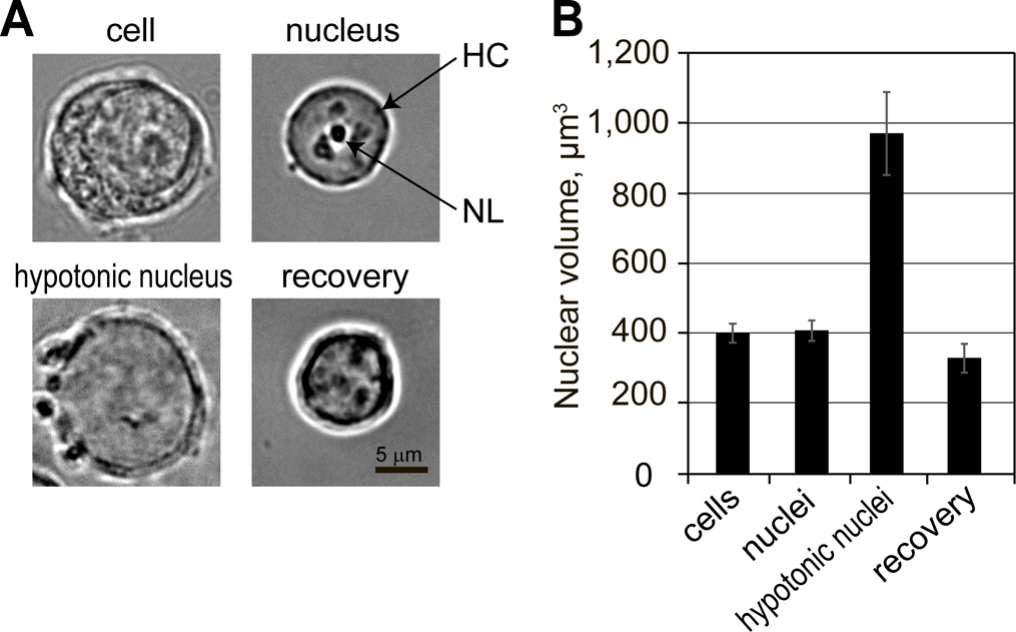
Expansion of nuclei under hypotonic conditions and recovery in the presence of a crowding agent. (A) Phase contrast images of the nucleus inside the cell (cell), the nucleus isolated in isotonic conditions (nucleus), the nucleus transferred into a law salt buffer (hypotonic nucleus) and the nucleus placed in a law salt buffer with a subsequent addition of 12.5% PEG 8 kDa (recovery). NL—nucleolus, HC—heterochromatin. (B) Mean volume of nuclei under the above-described conditions calculated based on their linear size measurement. The error bars represent SEM for 25 measurements.

**Fig 3:**
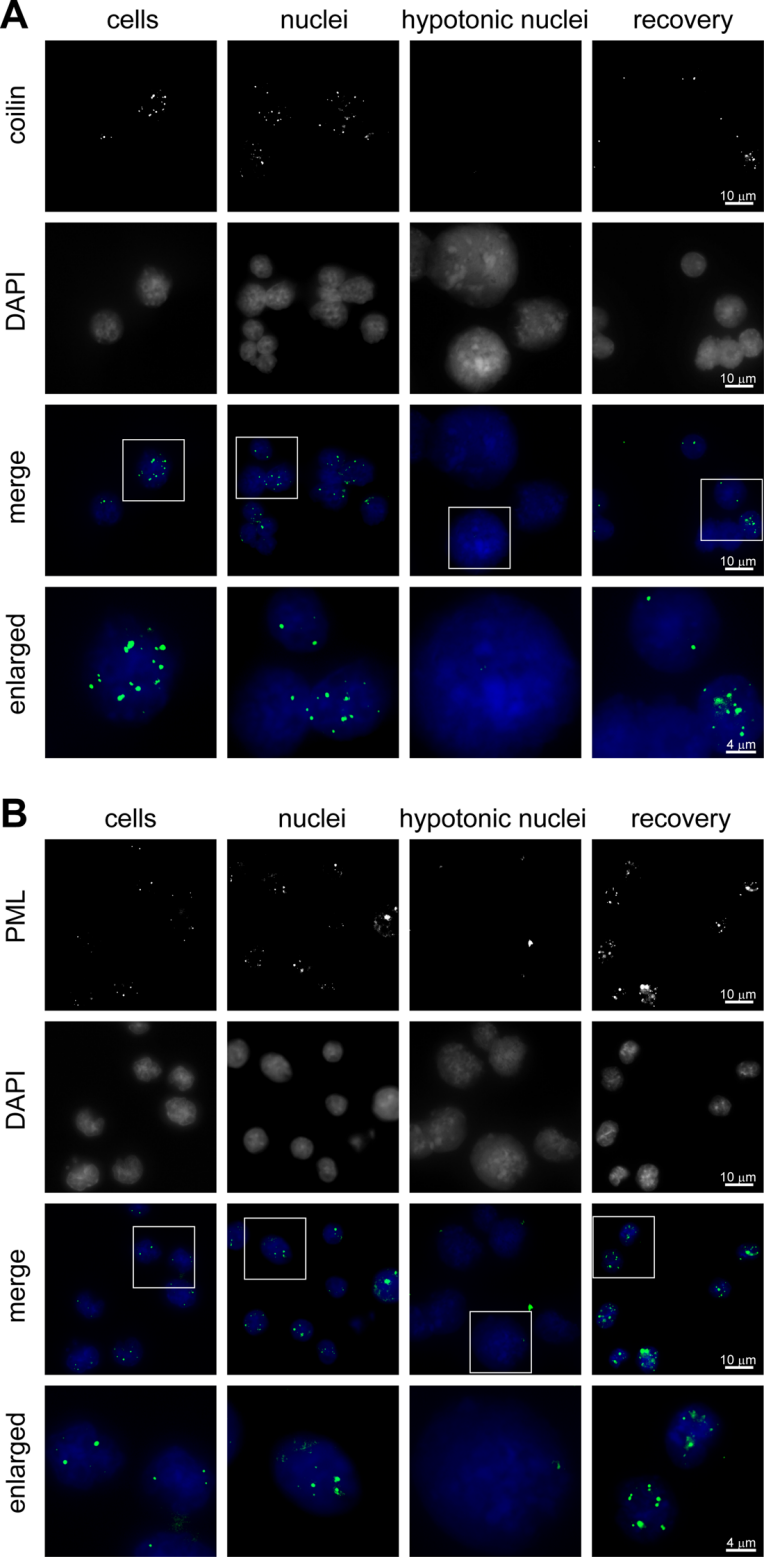
Disassembly of Cajal and PML bodies upon hypotonic expansion of the nuclei and reassembly of these compartments after restoration of the molecular crowding level. The cells and isolated nuclei fixed in different conditions were immunostained with antibodies against (A) coilin or (B) PML (the first row), and DNA was counterstained with DAPI (the second row). The third row shows superimposition of immunostaining (artificially colored green) and DAPI staining (artificially colored blue). The fourth raw shows the enlarged framed sections of the images presented in the third row.

Inspection of the “isotonic” nuclei under phase contrast demonstrated the presence of heterochromatin domains and nucleoli. These nuclei looked very similar to those in cells that were not lysed by digitonin (Fig 2A). The transfer of isolated nuclei into the hypotonic solution resulted in a significant expansion of the nuclear volume. The nuclei of MEL cells are known to have spherical shape [30]. Simple calculations based on measurements of nuclear diameter demonstrated that the nuclear volume increased more than 2 times from 410 +/- 30 to 970 +/- 120 μm^3^ (SEM, n = 25) (Fig 2B). An inspection of hypotonic nuclei under phase contrast revealed almost complete disappearance of the heterochromatin clusters and nucleoli (Fig 2A). To visualize PML and Cajal bodies, the cells and isolated nuclei were immunostained with antibodies that recognize PML and coilin, respectively, which are characteristic components of these compartments [6]. Both Cajal and PML bodies were clearly visible in cells and nuclei isolated in isotonic buffer but were not detectable after expansion of nuclei in hypotonic buffer (Fig 3).

The key observation made in the above-cited study by R. Hancock [15], was that upon the addition of an inert crowding agent (PEG 8 kDa) to a suspension of expanded nuclei in hypotonic buffer, the average size of the nuclei decreased to the initial value, and the nucleoli, PML and Cajal bodies were restored. As shown in Figs 2 and 3, this observation was fully reproduced in our model system. It should be noted that concentration of PEG 8 kDa that is sufficient to restore nuclear compartments under hypotonic conditions was determined experimentally in the above-cited study by R. Hancock [15]. It is difficult to say whether, after addition of PEG 8 kDa to this concentration, the level of macromolecular crowding within hypotonic nuclei matches or exceeds that in nuclei isolated under isotonic conditions. It is of importance, however, that the addition of PEG 8 kDa to a concentration higher than 12.5% results in a decrease of nuclear volume below the value observed in isotonic conditions ([15, 31], and data not shown).

**Fig 4:**
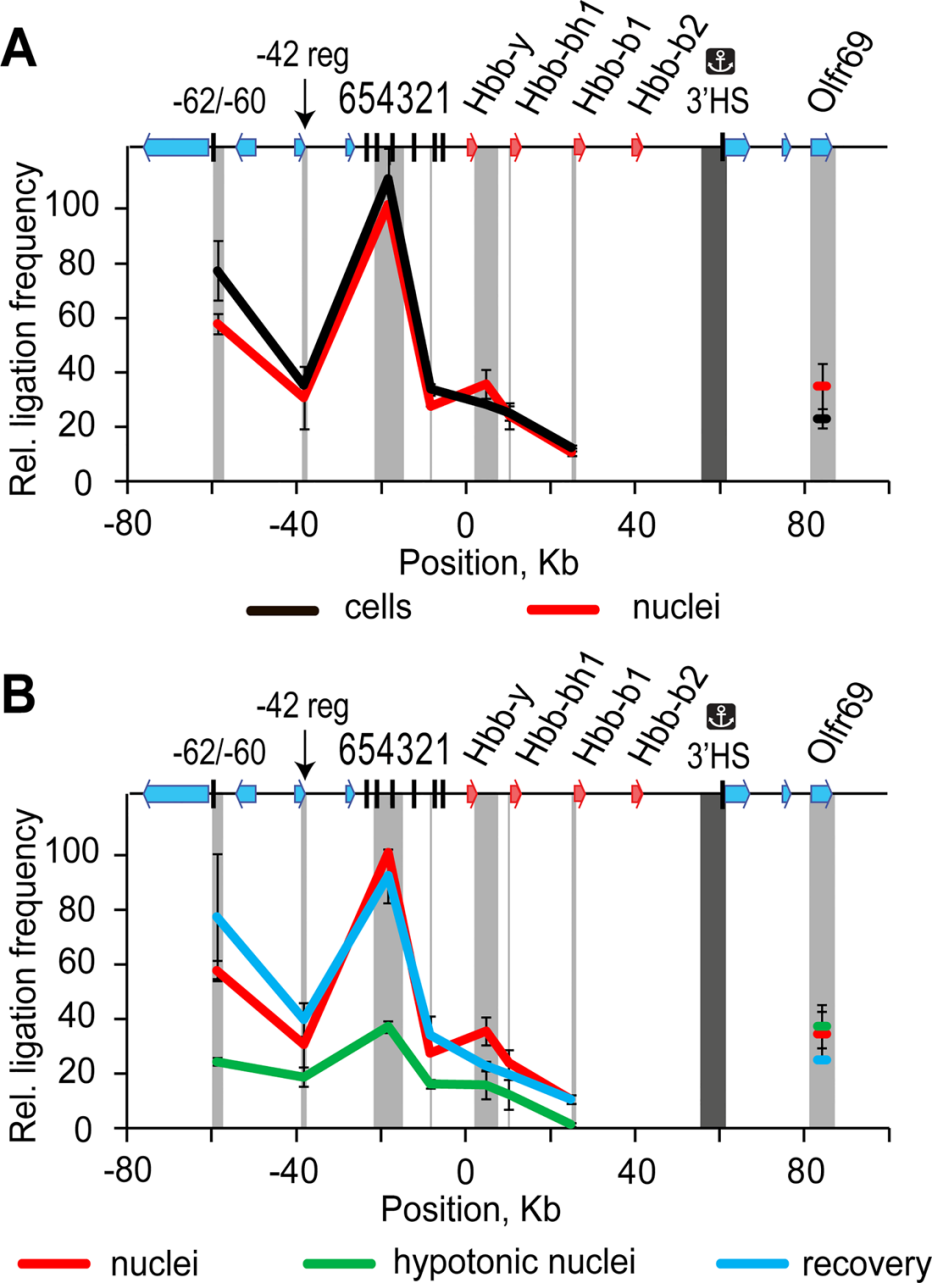
Frequencies of ligation of the fragment harboring the 3’-insulator of the β-globin gene domain with several selected fragments of the domain in MEL cells and isolated nuclei of these cells kept under different ionic and crowding conditions. (A) Results of 3C analysis performed on intact cells (black line) and nuclei isolated in isotonic conditions (red line). (B) Results of 3C analysis performed on nuclei isolated in isotonic conditions (red line), nuclei expanded in hypotonic buffer (green line) and nuclei incubated in hypotonic buffer with the subsequent addition of 12.5% PEG 8 kDa (blue line). A map of the domain is shown on the top of both graphs, with β-globin genes, olfactory receptor genes and DNase I hypersensitive sites shown by red arrows, blue arrows and black vertical lines, respectively. The 3’-insulator of the domain colocalizes with the 3’HS. The fragment positions are plotted on the horizontal axis. The scale is in kilobases, and according to GenBank entry NT_039433, the ‘0’ point corresponds to the start of the *Hbb*-y gene. The dark gray rectangle in the background of each graph shows the anchor fragment, and the light gray rectangles indicate test fragments. The ligation frequencies are plotted on the vertical axis; the frequency of ligation of the fragment bearing the 3’-insulator with the fragment bearing HS4/5 in isolated nuclei is set to 100. The error bars represent SEM for two independent experiments.

Having confirmed that we can trigger disassembly and reassembly of various compartments in nuclei of MEL cells by changing the level of molecular crowding, we focused our attention on the β-globin domain ACH. With the aim of finding out if entropy forces (depletion attraction forces) contribute to the juxtaposition of the β-globin domain regulatory elements, we compared the profiles of the spatial interaction of the 3’-insulator with the upstream regulatory elements of the domain (addressed in a previous study [32]) in cells, nuclei isolated in isotonic buffer, nuclei expanded in hypotonic buffer and nuclei expanded in hypotonic buffer and then returned to the normal size upon the addition of 12.5% PEG 8 kDa. It should be noted that in a standard 3C protocol fixed cells are used as the starting material [33]. Here, the formaldehyde fixation was performed on isolated nuclei. The results presented in Fig 4A demonstrate that the isolation of nuclei in isotonic buffer does not significantly influence the 3C profiles. Indeed, the 3C curves reflecting the profiles of spatial interactions of the anchor fragment (3’-insulator of the β-globin gene domain) with the upstream regulatory elements of the domain in non-lysed cells and in nuclei isolated in isotonic buffer, closely match each other. When the nuclei were first expanded in the hypotonic solution and then fixed with formaldehyde, a drastic loss of the 3C signal was observed (Fig 4B, green curve). This did not happen when PEG 8 kDa was added to a suspension of nuclei in the hypotonic buffer before formaldehyde fixation (Fig 4B, blue curve). To make sure that the reduced ligation frequencies observed in the experiments with hypotonic nuclei were not due to incomplete restriction enzyme digestion, we checked the digestion efficiency at all HindIII sites tested and found it to be sufficiently high both for hypotonic and “isotonic” or recovered nuclei (>85%, Figure A in S1 File). Moreover, judging by the level of circularization of the anchor restriction fragment and the fragment bearing *Hbb-b1* promoter (Figure B in S1 File), an overall efficiency of ligation was not affected either. This gives reason to believe that our 3C results reflect a change in the 3D arrangement of the ACH in hypotonic nuclei rather than differences in the efficiency of the 3C procedure.

The behavior of the β-globin domain ACH in response to reversible expansion of the nuclear volume closely resembled the behavior of various nuclear compartments. The spatial interactions between the remote regulatory elements of the β-globin domain were drastically decreased upon expansion of a nuclear volume in the hypotonic solution and were restored after addition of a crowding agent (PEG 8 kDa). Hence, the molecular links between the components of the β-globin domain ACH, such as dimeric or oligomeric complexes of proteins bound to different target sequences on DNA [34–36], are rather weak and their integrity depends on the “aid” of crowding forces. More importantly, the juxtaposition of the remote regulatory elements of the β-globin gene domain is likely mediated by the mode of chromatin fiber packaging, which can be reversibly changed depending on ionic strength and the level of molecular crowding. Otherwise, it is difficult to explain how the initial configuration of the domain, including juxtaposition of the remote regulatory elements, is quickly reestablished upon addition of a crowding agent to a suspension of nuclei in a hypotonic buffer.

It is not presently clear how contacts between remote genomic elements are established in chromatin context [20]. It might be proposed that in the course of erythroid cell differentiation, remote regulatory elements of the β-globin gene domain are repositioned to the same location by an active process (for example, by Pol II mediated transfer [37]) and are held together because of formation of a multicomponent complex. It should then be expected that once this complex is disassembled, the chromatin fiber would adopt the more favorable configuration and would keep this configuration unless the active process of ACH assembly is repeated from the beginning. However, any active biological processes of establishing communication between remote regulatory elements can hardly occur in isolated nuclei kept in the absence of NTPs for 20 min at 0°C (these were the conditions of incubation of the hypotonic nuclei with PEG 8 kDa before formaldehyde fixation). Thus, the only possibility to explain restoration of the 3C signal observed in our experiments after the addition of PEG to a suspension of nuclei in a hypotonic buffer is to assume that under these conditions, the most favorable configuration of the chromatin fiber is the one that brings together the components of the β-globin domain ACH. Restoration of this particular configuration can be guided by specific profiles of histone modifications or association with HMG and other non-histone proteins that have been established during differentiation and are not affected by the decrease of the crowding forces upon nuclear expansion in a hypotonic buffer. To this end, it is of note that decompaction of heterochromatin clusters in living cells placed in hypotonic medium did not result in a loss of heterochromatin-specific histone modifications. Furthermore, super-compaction of euchromatic regions under hypertonic conditions did not correlate with acquisition of these modifications [38]. Crowding forces are known to support self-association of polynucleosomal chains [39]. Thus, it is not surprising that upon a decrease in the level of macromolecular crowding, the chromatin fiber adopts a new configuration. Nevertheless, it possesses a kind of spatial memory that is based on physical laws and allows the initial configuration to be reestablished upon restoration of the crowding forces. It is currently not clear whether enhancers and promoters of beta-globin genes are indeed assembled in a common complex. The other possibility is that they are located in spatial proximity because of the particular folding of a chromatin fiber [18, 25]. As both the folding of a chromatin fiber and the stability of supramolecular complexes can be influenced by the level of molecular crowding, our results fit both the active chromatin hub [22] and the expression hub [40] models. In fact, these models are not mutually exclusive. Being brought into spatial proximity by the specific folding of a chromatin fiber, enhancers and promoters may participate in the formation of supra-molecular complexes via interaction of linked proteins. Interaction of molecules possessing complementary surfaces is especially strongly stabilized by entopic forces [41] that thus may support self-association of CTCF and oligomerization of transcription factors (such as Sp1 [35, 36] and Ldb1 [34]) bound to remote genomic sites.

## Materials and Methods

### Cell culture

The murine adult erythroleukemia cell line MEL (MEL-745A cl. DS19, CABRI:DSMZ_MUTZ ACC 501) was cultured in RPMI–1640 medium, containing 10% fetal bovine serum (FBS), 100 units/ml penicillin and 100 μg/ml streptomycin in 5% CO_2_ and 37°C. For induction of the globin gene transcription, cells were incubated with 2% dimethyl sulfoxide (DMSO) for 72 h, as described previously [42]. Transcription of the adult beta-globin genes *Hbb-b1* and *Hbb-b2* is significantly stimulated upon induction (Figure C in S1 File).

### Isolation of nuclei

The cells were washed once in phosphate buffered saline (PBS) and then resuspended in ice cold isotonic nuclear buffer, INB [which consisted of 10 mM Hepes-NaOH (pH 7.5), 5 mM NaCl, 140 mM KCl, 300 μM MgCl_2_, 1 mM dithiothreitol (DTT), 0.1 mM phenylmethanesulfonylfluoride (PMSF)], supplemented with 50 μg/ml digitonin (Sigma), at a concentration of 6×10^7^ cells/ml, and incubated on ice for 10 min. The nuclei were released by 50 gentle hand strokes of a small clearance pestle in a 2 ml Dounce homogenizer (Kimble Chase).

### Expansion of the nuclei in a solution with low ionic strength, recovery of the nuclear volume by a crowding agent and fixation of isolated nuclei in different conditions

The suspension of the nuclei in INB was diluted by the addition of three volumes of INB without digitonin and three aliquots (each containing 2×10^7^ nuclei) were collected. The nuclei from each aliquot were sedimented at low speed (200 g) and resuspended in either ice-cold INB (one aliquot) or ice-cold hypotonic nuclear buffer, HNB [which consisted of 10 mM Hepes-NaOH (pH 7.5), 5 mM NaCl, 300 μM MgCl_2_, 1 mM DTT, and 0.1 mM PMSF] (two aliquots). All three samples were incubated for 10 min on ice followed by the addition to two of these samples, one in INB and one in HNB, of an equal volume of warm (37°C) fixation solution, which was 4% formaldehyde prepared in INB or HNB, respectively. Fixation was conducted for 15 min at room temperature. The second (non-fixed) aliquot of nuclei in HNB was mixed (carefully but thoroughly) with a 1/3 volume of cold 50% 8 kDa polyethylene glycol (PEG) solution in the same buffer. After an additional 20 min incubation on ice, a warm 4% solution of formaldehyde in HNB supplemented with 12.5% PEG 8 kDa was added, and the nuclei were fixed for 15 min at room temperature. The fixed nuclei in all three cases were harvested and washed in PBS supplemented with 125 mM glycine.

### Optical imaging and nuclear volume assessment

To assess the diameters of the nuclei, phase-contrast images were used. Approximate nuclear volumes were calculated based on an assumption that the erythroblast’s nuclei have a spherical shape. Immunostaining of the cells and isolated nuclei were performed as previously described^25^. The following primary antibodies were used: mouse anti-PML (Millipore, MAB3738) and mouse anti-coilin (Abcam, ab87913). The primary antibodies bound to antigens were visualized using Alexa Fluor 488-conjugated secondary antibodies: rabbit anti-mouse AF488 (Invitrogen, A11059). The DNA was counterstained with 4’,6-diamidino-2-phenylindole (DAPI). The results were inspected using a Zeiss Axio Scope.A1 microscope equipped with a 100× EC Plan-Neofluar 100×/1.30 Oil-immersion objective.

### Western blot

Equal aliquots of cells or nuclei suspension were lysed in lysis buffer [50mM Tris-HCl, pH 8.0; 5 mM EDTA, 1% SDS, 0.1 mM PMSF and protease inhibitors cocktail (Roche, 04693116001)] for 10 minutes, then lysates were sonicated (VirTis VirSonic 100 sonicator, setting 20, 30 second pulse). Equal volumes of lysates were electrophoresed in 8%, 12% or 15%SDS-polyacrylamide gel and transferred onto polyvinylidene difluoride membranes (Hybond-P, Amersham Biosciences). The membranes were blocked with 2% Amersham ECL Blocking Agent (Amersham Biosciences) in PBS containing 0.1% Tween 20 (PBS-T) and subsequently incubated with primary antibodies overnight at 4°C. The following primary antibodies were used: mouse anti-PML (Millipore, MAB3738), rabbit anti-GATA–1 (Santa Cruz, sc13053), rabbit anti-Topoisomerase I (Abcam, ab3825), and rabbit anti-histone H3 (Active Motif, 39163). After three washing with PBS-T at room temperature, the membrane was further incubated with appropriate horseradish peroxidase (HRP)-conjugated secondary antibodies for 1 hour, followed by five washings with PBS-T. The following secondary antibodies were used: HRP-conjugated sheep anti-mouse IgG (GE Healthcare, NA931) and HRP-conjugated donkey anti-rabbit IgG (GE Healthcare, NA934V). Protein bands were visualized with Pierce ECL Plus substrate (Pierce). Protein band quantification was carried out using ImageJ software.

### Chromosome conformation capture (3C)

The 3C procedure was performed as described elsewhere [21, 25]. In a standard experiment 5×10^6^ fixed cells or nuclei were taken. The Hind III restriction enzyme was used for DNA digestion. The ligation products were analyzed by TaqMan real-time PCR. A random ligation standard was generated using a bacterial artificial chromosome carrying the murine β-globin gene locus along with flanking sequences (BAC clone RP24-79I7, CHORI BACPAC Resources Center). The sequences of primers and TaqMan probes are presented in Table A in S1 File.

## Supporting Information

S1 File Sequences of primers and TaqMan probes used for 3C analysis and PCR-stop analysis (**Table A**). Sequences of primers used for RT-qPCR analysis (**Table B**). Efficiency of cleavage of the beta-globin gene locus at different HindIII sites involved in the 3C analysis (**Figure A**). Frequencies of circularization (self-ligation) of the anchor restriction fragment bearing 3’-insulator and the fragment bearing *Hbb-b1* promoter (**Figure B**). Transcription activity of embryonic beta-globin genes *Hbb-y* and *Hbb-bh1* and adult beta-globin genes *Hbb-b1* and *Hbb-b2* in MEL cells before and after induction (**Figure C**).
(PDF)

## Abbreviations

3C: chromosome conformation capture
ACH: active chromatin hub
LCR: locus control region
HS: DNase I hypersensitive site
PEG: polyethylene glycol

## References

[1] Carmo-Fonseca M, Rino J. RNA seeds nuclear bodies. Nat Cell Biol. 2011;13(2):110–2. 10.1038/ncb0211-110 . 21283118

[2] Mao YS, Zhang B, Spector DL. Biogenesis and function of nuclear bodies. Trends Genet. 2011;27(8):295–306. 10.1016/j.tig.2011.05.006 21680045 PMC3144265

[3] Sleeman JE, Trinkle-Mulcahy L. Nuclear bodies: new insights into assembly/dynamics and disease relevance. Curr Opin Cell Biol. 2014;28C:76–83. 10.1016/j.ceb.2014.03.004 . 24704702

[4] Marenduzzo D, Micheletti C, Cook PR. Entropy-driven genome organization. Biophys J. 2006;90(10):3712–21. Epub 2006/02/28. 10.1529/biophysj.105.077685 16500976 PMC1440752

[5] Rajendra TK, Praveen K, Matera AG. Genetic analysis of nuclear bodies: from nondeterministic chaos to deterministic order. Cold Spring Harb Symp Quant Biol. 2010;75:365–74. 10.1101/sqb.2010.75.043 . 21467138 PMC4062921

[6] Dundr M. Nuclear bodies: multifunctional companions of the genome. Curr Opin Cell Biol. 2012;24(3):415–22. 10.1016/j.ceb.2012.03.010 22541757 PMC3372688

[7] Asakura S, Oosawa F. Interaction between particles suspended in solutions of macromolecules. J Polym Sci. 1958;33:183–92.

[8] Kim JS, Szleifer I. Depletion Effect on Polymers Induced by Small Depleting Spheres. J Phys Chem C. 2010;114 20864–9.

[9] Hancock R. Internal organisation of the nucleus: assembly of compartments by macromolecular crowding and the nuclear matrix model. Biol Cell. 2004;96(8):595–601. . 15519694 10.1016/j.biolcel.2004.05.003

[10] Hancock R. The crowded nucleus. International review of cell and molecular biology. 2014;307:15–26. 10.1016/B978-0-12-800046-5.00002-3 . 24380591

[11] Cho EJ, Kim JS. Crowding effects on the formation and maintenance of nuclear bodies: insights from molecular-dynamics simulations of simple spherical model particles. Biophys J. 2012;103(3):424–33. 10.1016/j.bpj.2012.07.007 22947858 PMC3414890

[12] Kim JS, Szleifer I. Crowding-induced formation and structural alteration of nuclear compartments: insights from computer simulations. International review of cell and molecular biology. 2014;307:73–108. 10.1016/B978-0-12-800046-5.00004-7 . 24380593

[13] Ellis RJ. Macromolecular crowding: obvious but underappreciated. Trends Biochem Sci. 2001;26(10):597–604. Epub 2001/10/09. . 11590012 10.1016/s0968-0004(01)01938-7

[14] Richter K, Nessling M, Lichter P. Macromolecular crowding and its potential impact on nuclear function. Biochim Biophys Acta. 2008;1783(11):2100–7. 10.1016/j.bbamcr.2008.07.017 . 18723053

[15] Hancock R. A role for macromolecular crowding effects in the assembly and function of compartments in the nucleus. J Struct Biol. 2004;146(3):281–90. Epub 2004/04/22. 10.1016/j.jsb.2003.12.008 . 15099570

[16] Bickmore WA. The spatial organization of the human genome. Annual review of genomics and human genetics. 2013;14:67–84. 10.1146/annurev-genom-091212-153515 . 23875797

[17] Cavalli G, Misteli T. Functional implications of genome topology. Nat Struct Mol Biol. 2013;20(3):290–9. 10.1038/nsmb.2474 . 23463314 PMC6320674

[18] Razin SV, Gavrilov AA, Ioudinkova ES, Iarovaia OV. Communication of genome regulatory elements in a folded chromosome. FEBS Lett. 2013;587(13):1840–7. 10.1016/j.febslet.2013.04.027 . 23651551

[19] de Graaf CA, van Steensel B. Chromatin organization: form to function. Curr Opin Genet Dev. 2013;23(2):185–90. 10.1016/j.gde.2012.11.011 . 23274160

[20] Ulianov SV, Gavrilov AA, Razin SV. Nuclear compartments, genome folding, and enhancer-promoter communication. International review of cell and molecular biology. 2015;315:183–244. 10.1016/bs.ircmb.2014.11.004 . 25708464

[21] Tolhuis B, Palstra RJ, Splinter E, Grosveld F, de Laat W. Looping and interaction between hypersensitive sites in the active beta-globin locus. Mol Cell. 2002;10(6):1453–65. . 12504019 10.1016/s1097-2765(02)00781-5

[22] de Laat W, Grosveld F. Spatial organization of gene expression: the active chromatin hub. Chromosome Res. 2003;11:447–59. 12971721 10.1023/a:1024922626726

[23] de Laat W, Klous P, Kooren J, Noordermeer D, Palstra RJ, Simonis M, et al Three-dimensional organization of gene expression in erythroid cells. Curr Top Dev Biol. 2008;82:117–39. 10.1016/S0070-2153(07)00005-1 18282519

[24] Gavrilov AA, Chetverina HV, Chermnykh ES, Razin SV, Chetverin AB. Quantitative analysis of genomic element interactions by molecular colony technique. Nucleic Acids Res. 2014;42(5):e36 10.1093/nar/gkt1322 24369423 PMC3950710

[25] Gavrilov AA, Gushchanskaya ES, Strelkova O, Zhironkina O, Kireev II, Iarovaia OV, et al Disclosure of a structural milieu for the proximity ligation reveals the elusive nature of an active chromatin hub. Nucleic Acids Res. 2013;41:3563–75. 10.1093/nar/gkt067 . 23396278 PMC3616722

[26] Phillips-Cremins JE, Sauria ME, Sanyal A, Gerasimova TI, Lajoie BR, Bell JS, et al Architectural protein subclasses shape 3D organization of genomes during lineage commitment. Cell. 2013;153(6):1281–95. 10.1016/j.cell.2013.04.053 23706625 PMC3712340

[27] Rao SS, Huntley MH, Durand NC, Stamenova EK, Bochkov ID, Robinson JT, et al A 3D map of the human genome at kilobase resolution reveals principles of chromatin looping. Cell. 2014;159(7):1665–80. 10.1016/j.cell.2014.11.021 . 25497547 PMC5635824

[28] Adam SA, Sterne-Marr R, Gerace L. Nuclear protein import using digitonin-permeabilized cells. Methods Enzymol. 1992;219:97–110. . 1488017 10.1016/0076-6879(92)19013-v

[29] Bickmore WA, Sutherland HG. Addressing protein localization within the nucleus. EMBO J. 2002;21(6):1248–54. 10.1093/emboj/21.6.1248 11889031 PMC125932

[30] Hepperger C, Otten S, von Hase J, Dietzel S. Preservation of large-scale chromatin structure in FISH experiments. Chromosoma. 2007;116(2):117–33. 10.1007/s00412-006-0084-2 17119992 PMC1824788

[31] Hancock R, Hadj-Sahraoui Y. Isolation of cell nuclei using inert macromolecules to mimic the crowded cytoplasm. PLoS One. 2009;4(10):e7560 Epub 2009/10/24. 10.1371/journal.pone.0007560 19851505 PMC2762040

[32] Splinter E, Heath H, Kooren J, Palstra RJ, Klous P, Grosveld F, et al CTCF mediates long-range chromatin looping and local histone modification in the beta-globin locus. Genes Dev. 2006;20(17):2349–54. . 16951251 10.1101/gad.399506 PMC1560409

[33] Miele A, Dekker J. Mapping cis- and trans- chromatin interaction networks using chromosome conformation capture (3C). Methods Mol Biol. 2009;464:105–21. 10.1007/978-1-60327-461-6_7 18951182 PMC3874836

[34] Song SH, Hou C, Dean A. A positive role for NLI/Ldb1 in long-range beta-globin locus control region function. Mol CeLL. 2007;28(5):810–22. 10.1016/j.molcel.2007.09.025 18082606 PMC2195932

[35] Li R, Knight JD, Jackson SP, Tjian R, Botchan MR. Direct interaction between Sp1 and the BPV enhancer E2 protein mediates synergistic activation of transcription. Cell. 1991;65(3):493–505. Epub 1991/05/03. . 1850324 10.1016/0092-8674(91)90467-d

[36] Su W, Jackson S, Tjian R, Echols H. DNA looping between sites for transcriptional activation: self-association of DNA-bound Sp1. Genes Dev. 1991;5(5):820–6. Epub 1991/05/01. . 1851121 10.1101/gad.5.5.820

[37] Larkin JD, Papantonis A, Cook PR, Marenduzzo D. Space exploration by the promoter of a long human gene during one transcription cycle. Nucleic Acids Res. 2013;41(4):2216–27. Epub 2013/01/11. 10.1093/nar/gks1441 23303786 PMC3575846

[38] Walter A, Chapuis C, Huet S, Ellenberg J. Crowded chromatin is not sufficient for heterochromatin formation and not required for its maintenance. J Struct Biol. 2013;184(3):445–53. Epub 2013/10/23. 10.1016/j.jsb.2013.10.004 . 24145303

[39] Hancock R. Self-association of polynucleosome chains by macromolecular crowding. European biophysics journal: EBJ. 2008;37(6):1059–64. Epub 2008/02/09. 10.1007/s00249-008-0276-1 . 18259740

[40] Kosak ST, Groudine M. Form follows function: The genomic organization of cellular differentiation. Genes Dev. 2004;18(12):1371–84. . 15198979 10.1101/gad.1209304

[41] Kinoshita M, Oguni T. Depletion effects on the lock and key steric interactions between macromolecules. Chemical Physics Letters. 2002;351(1–2):79–84.

[42] Ganguly S, Skoultchi AI. Absolute rates of globin gene transcription and mRNA formation during differentiation of cultured mouse erythroleukemia cells. J Biol Chem. 1985;260(22):12167–73. . 3862667

