# Supplementary material for: The Role of Crowding Forces in Juxtaposing β-Globin Gene Domain Remote Regulatory Elements in Mouse Erythroid Cells
Source: PLoS One. 2015 Oct 5;10(10):e0139855. doi: 10.1371/journal.pone.0139855 (PMC4593578; doi:10.1371/journal.pone.0139855)
Supplement: S1 File — Sequences of primers and TaqMan probes used for 3C analysis and PCR-stop analysis (Table A). Sequences of primers used for RT-qPCR analysis (Table B). Efficiency of cleavage of the beta-globin gene locus at different HindIII sites involved in the 3C analysis (Figure A). Frequencies of circularization (self-ligation) of the anchor restriction fragment bearing 3’-insulator and the fragment bearing Hbb-b1 promoter (Figure B). Transcription activity of embryonic beta-globin genes Hbb-y and Hbb-bh1 and adult beta-globin genes Hbb-b1 and Hbb-b2 in MEL cells before and after induction (Figure C). (PDF) [file pone.0139855.s001.pdf]

Supporting Information, S1 File

Supplementary Tables

Table A. Sequences of primers and TaqMan probes used for 3C analysis and PCR-stop analysis.

Test fragment	Primer/TaqMan set (5'-3')
HS -62/-60	a/s GGGTGTGGGTATTTGTAAGAG<br>s* GGTCTACGAACATGCTGTAGTT
-42 region	a/s ATGAACAAGTTTCATGGGG<br>s* TAGTCTGGGCTAGTGACAGAA
HS4/5	a/s TTCAAGTTCTCATCCTTCACTG<br>s* GTACACTGTGCAGGTGATTTTG
HS2	a/s GCAGCTTCCTCATTTAGCA<br>s* CCTCCTTCACTCTATATCCAACT
Hbb-y – Hbb-bh1 intergenic region	s CCCCCAAGACTCTGCTGAAG<br>a/s* ATTCGTGAGATGGCAAATATGTAC
Hbb-bh1 promoter	a/s ATAAGTGTAGCTGCCTGGTG<br>s* AAACACACAAAAAGTGAAAACAGAG
Hbb-b1 promoter	s AATCGCTGCTCCCCCTCACT<br>a/s* GATGGGAAGTAAATAACCAGCTTAAT
Hbb-b1 promoter (circularization)	a/s CAACACATTTGCTCAATCAACTACT<br>TM FAM-ACCAAAGAAAGAGGAAA(T-BHQ1)GACAACACAGAACA-PO_4
Hbb-b1 promoter (internal control)	s* TCAGTAGTTGATTGAGCAAATGTGT<br>a/s* TCGGTGATGACAAGCATATTTCT
3'-insulator (anchor)	s TCCTTGCTTTTACTCTTTCTCC<br>TM FAM-AGCTTCCTT(T-BHQ1)GAACATAACTTTGCACTTACTTGTTTCTG-PO_4<br>a/s* ACTTGACTAAGTTAGGTGTTGTACTCT
3'-insulator (circularization)	a/s TCCAACTCAATTCTTCAACGA
Olfr69	s ACTGCACTGTCTTCCAAATCACT<br>a/s* GGGGCAAACAAAATGACAAGT

s, sense primers, a/s, antisense primers (with respect to the direction of transcription of beta-globin genes), TM, TaqMan probes. Asterisks denote PCR-stop primers which were used in combination with the corresponding 3C primer.

Table B. Sequences of primers used for RT-qPCR analysis.

Gene	Primer set (5'-3')
Hbb-y	s TGACAAATGGAGGCTTGTTCTT<br>a/s CCTGTCGTTATTCTCATTCTACAGTT
Hbb-bh1	s CATGCTCATGTGTTCATTATGGA<br>a/s TATTAAGATTTCTACAACGGTTACTGAC
Hbb-b1/2	s AGTCTGATGGGCACCTCCTG<br>a/s GACTGCTCCCTAGAATCGCTT
Olfr69	s GGCAAAGCACATTTACCTGAT<br>a/s ACACTTGTTCCCTGAGATGGTT
GAPDH	s AATCAAAGCGGACTTACAGAGG<br>a/s TTTCAGAACCACCATGACTCAG

s, sense primers, a/s, antisense primers (with respect to the direction of transcription of beta-globin genes or *GAPDH*).

Supplementary Figures

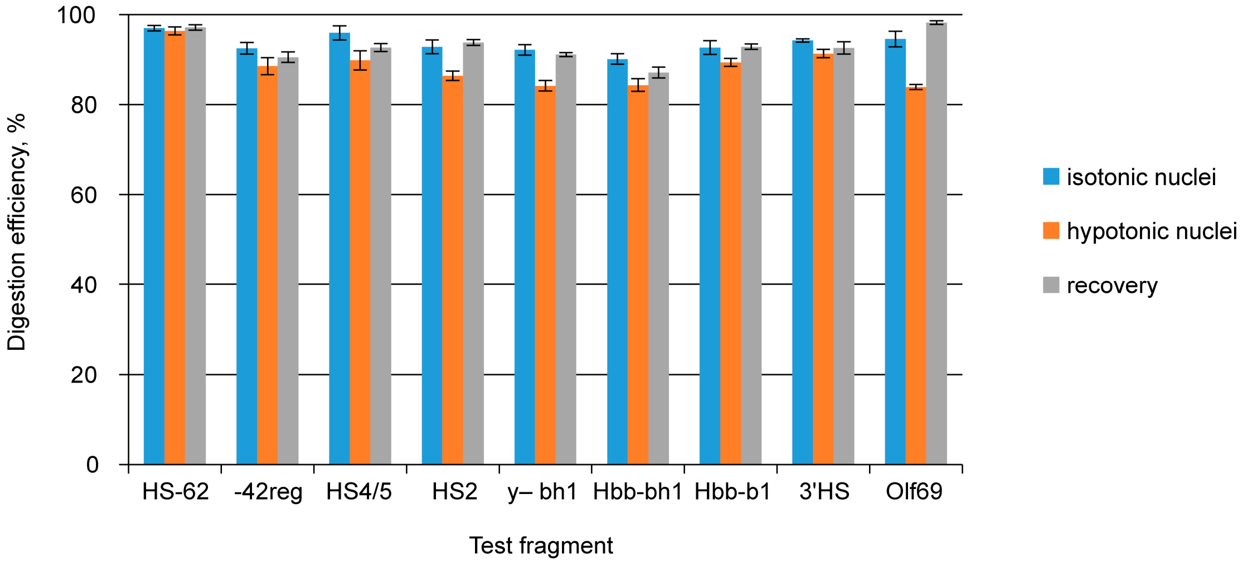

Figure A. Efficiency of cleavage of the beta-globin gene locus at different HindIII sites involved in the 3C analysis. DNA was purified after the restriction digestion step followed by EvaGreen qPCR analysis with amplicons spanning restrictions sites of interest (PCR-stop amplicons) and a control amplicon in the inner portion of a restriction fragment (internal

amplicon). The digestion efficacy (DE) was calculated using the following equation: DE(%)=[1–A(PCR-stop)/A(internal)]*100, where A(PCR-stop) – PCR signal observed with a PCR-stop amplicon, A(internal) – PCR signal observed with the internal amplicon located within the HindIII-fragment bearing *Hbb-b1* promoter. The sequences of primers used for qPCR are presented in Table A in S1 File. The error bars represent SEM for two independent experiments.

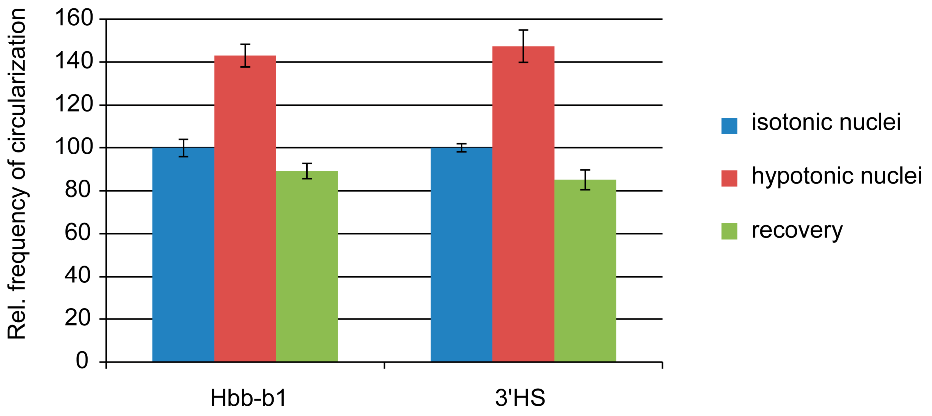

Figure B. Frequencies of circularization (self-ligation) of the anchor restriction fragment bearing 3'-insulator and the fragment bearing *Hbb-b1* promoter. The frequency of ligation observed in the experiments with "isotonic" nuclei was taken equal to 100, and the data for hypotonic and recovered nuclei were normalized accordingly. The sequences of primers and TaqMan probes used for the analysis of ligation frequencies are presented in Table A in S1 File. The error bars represent SEM for two independent experiments.

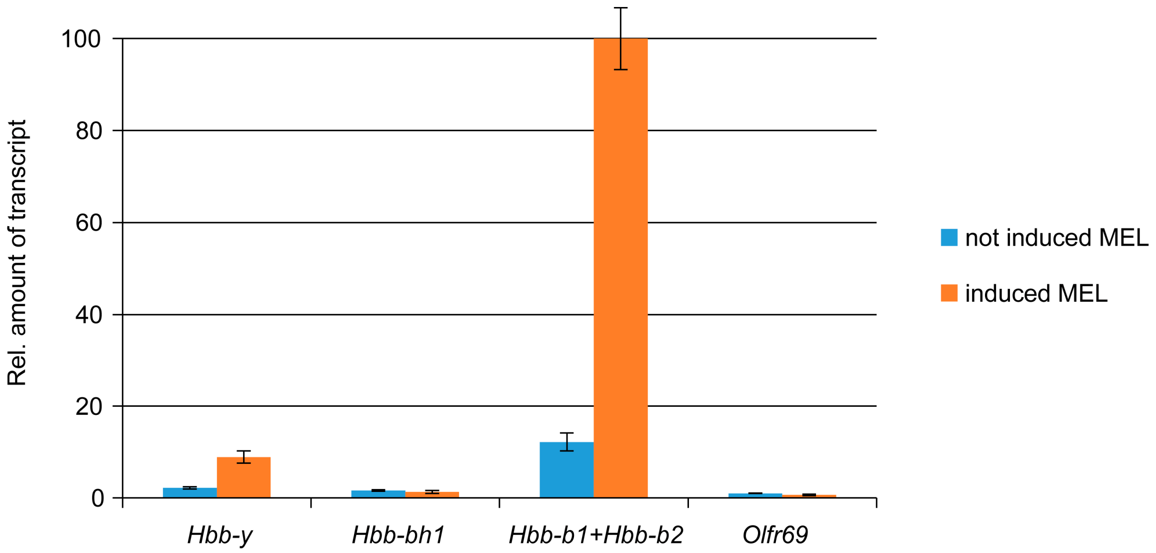

Figure C. Transcription activity of embryonic beta-globin genes *Hbb-y* and *Hbb-bh1* and adult beta-globin genes *Hbb-b1* and *Hbb-b2* in MEL cells before and after induction, as

determined by reverse transcription followed by EvaGreen qPCR with primers to intronic regions. The sequences of primers used for qPCR are presented in Table B in S1 File. Note that due the high sequence similarity between *Hbb-b1* and *Hbb-b2* genes our analysis determines the total amount of transcripts of these two genes. For a negative control, the transcription level of the olfactory receptor gene *Olfr69* was evaluated. The raw data were normalized to the amount of GAPDH transcript. The amount of transcripts of adult beta-globin genes (*Hbb-b1* + *Hbb-b2*) in induced MEL cells was taken equal to 100. The error bars represent SEM for two independent experiments.
